# Children With Noncritical Infections Have Increased Intestinal Permeability, Endotoxemia and Altered Innate Immune Responses

**DOI:** 10.1097/INF.0000000000002311

**Published:** 2019-07-01

**Authors:** Jonathan P. Sturgeon, Claire D. Bourke, Andrew J. Prendergast

**Affiliations:** * Centre for Genomics and Child Health, Blizard Institute, Queen Mary University of London, London, United Kingdom; † Department of Paediatrics, Royal London Hospital, Barts Health NHS Trust, London, United Kingdom

**Keywords:** child, gut permeability, innate immunity, monocytes, lipopolysaccharide

## Abstract

**Background:**

Children with critical illness have increased intestinal permeability and a period of immunoparalysis, mediated by elevated circulating endotoxin. Whether children with less severe infections have similar changes is uncertain.

**Methods:**

We conducted a proof-of-concept pilot study, enrolling children 6–59 months of age hospitalized for noncritical infections (cases, n = 11) and noninfected controls (n = 19). Intestinal permeability was measured by lactulose–mannitol recovery. Plasma endotoxin, blood monocyte and neutrophil immunophenotypes and cytokine elaboration following 24-hour whole-blood culture with antigens targeting distinct innate pathogen recognition receptor signaling pathways were evaluated.

**Results:**

Cases had higher intestinal permeability and plasma endotoxin levels than controls. Among cases versus controls, fewer monocytes expressed human leukocyte antigen DR isotype (HLA-DR) (87.1% vs. 96.4%, *P* = 0.001), and more expressed CD64 (99.6% vs. 97.6%, *P* = 0.041). Following zymosan stimulation of whole blood, cases versus controls produced less interleukin 1 beta (IL-1β) (median 1101 vs. 2604 pg/mL, *P* = 0.048) and tumor necrosis factor alpha (TNF-α) (2342 vs. 5130 pg/mL, *P* = 0.031). Children with higher (≥0.1 endotoxin unit (EU)/mL) versus lower (<0.1 EU/mL) circulating endotoxin had fewer monocytes expressing CD86 (69.8% vs. 92.4%, *P* = 0.003) and less expression of CD64 following 24-hour zymosan stimulation (median fluorescence intensity (MFI) 1514 vs. 2196, *P* = 0.022).

**Conclusions:**

Children hospitalized with noncritical infections had increased intestinal permeability, endotoxemia and altered monocyte phenotype and function. Collectively, these changes are typical of immunoparalysis seen in children with critical illness and may increase the risk of subsequent infections.

Children with critical illness, such as sepsis, subsequently have a period of reversible immune dysfunction.^1^ This state of immunoparalysis is characterized by reduced monocyte function, including lower human leukocyte antigen DR isotype (HLA-DR) expression and reduced tumor necrosis factor alpha (TNF-α) production upon restimulation, increased risk of secondary infections and elevated mortality.^1^ A potent mediator of this process is lipopolysaccharide (LPS),^2^ also called endotoxin, a highly immunogenic component of the outer membrane of Gram-negative bacteria. “LPS tolerance” can reduce the proinflammatory response to a subsequent immune challenge^3^ through alterations in metabolic and epigenetic pathways that drive innate immune cell reprograming.^4^

Elevated circulating levels of LPS (endotoxemia) are detectable even in Gram-positive infections,^5^ and noninfectious conditions like trauma,^6^ obesity,^7^ severe autism^8^ and cardiac arrest.^9^ Thus, endotoxaemia is not necessarily an indicator of infection. The source of circulating LPS remains uncertain, but the intestinal tract represents a plausible site because it contains a large number of commensal Gram-negative bacteria as part of the gut microbiota.^10^ The gut is fundamentally altered during critical illness, with overgrowth of bacterial species in the upper intestine, dysbiosis of the microbiota in the lower intestine and loss of gut barrier function.^11^ With increased intestinal permeability, bacteria and microbial products can translocate to mesenteric lymph nodes and to the bloodstream, exposing immune cells directly to bacterial antigens, including LPS. Whether this process drives immunoparalysis and increased susceptibility to subsequent infections in critical illness remains uncertain, but has been widely proposed by clinical studies^5,6^ and is supported by data from animal models.^12,13^

To date, immunoparalysis has been predominantly investigated in critical illness; however, it is possible that immune cell phenotype and function can also be modulated in the context of less severe infections. Epidemiologic data suggest that an episode of infectious illness increases the risk of subsequent infections of a child, and it has been suggested that immunoparalysis may be one mechanism underlying this observation.^14^ Furthermore, intestinal permeability can be increased during systemic,^15^ intestinal^16,17^ and nonintestinal infections.^18^ Given that the immune response to intravenously administered endotoxin can itself increase intestinal permeability in healthy adult volunteers,^19–23^ a reinforcing cycle may occur, with potential to affect both subsequent immune responses and intestinal permeability.

We therefore set out to investigate first whether children with noncritical infections have evidence of increased intestinal permeability, endotoxemia and innate immune dysfunction similar to the immunoparalysis of critical illness. Second, we investigated whether endotoxemia was associated with immune dysfunction by comparing innate immune cell phenotype and function between groups of children stratified by their circulating LPS levels, independently of their infection status.

## Materials and Methods

### Ethics

The study received ethical approval from UK National Health Service Health Research Authority Research Ethics Service through the Hampstead Research Ethics Committee (16/LO/2213). Participants were recruited following written informed consent from their parent or guardian.

### Study Cohort

Children 6–59 months of age were recruited from the Royal London Hospital between March and June 2017. Cases were recruited from children hospitalized for noncritical infections, which we defined as having a documented fever above 38°C and C-reactive protein (CRP), if measured for clinical management, >10mg/L, with no end-organ dysfunction. We chose broad criteria to identify children with a systemic febrile and inflammatory response to infection, regardless of the specific etiology, to reflect the wide range of infectious causes and diagnostic uncertainty when treating children with fever. Controls were defined as children who were either hospitalized or attending the day-care unit, were not acutely unwell, had no fever in the past week and, if measured, had CRP < 10 mg/L. Controls were therefore free of symptomatic infection (the main focus of this study), but did have noninfectious conditions requiring a day visit to hospital. The collection of urine and blood was carried out before any planned day-case procedure.

Cases and controls were excluded if they had gastrointestinal symptoms, defined as persistent vomiting or passage of 3 or more watery or loose stools in the previous 24 hours. Any children with known or suspected chronic inflammatory or gastrointestinal disease were excluded.

### Collection of Blood

Up to 5 mL of blood was collected from all participants by venepuncture into a glass sodium heparin tube (BD, Franklin Lakes, NJ). The duration of infectious symptoms before hospital admission varied at the time of sampling and was not standardized across participants. Although endotoxin concentrations are not reported by the manufacturer, random sampling of blood collection tubes after addition of 3 mL of endotoxin-free water revealed LPS concentrations <0.01 EU/mL. All other laboratory equipment was certified endotoxin free. Whole blood was used for assays as described below, then the remaining sample was centrifuged at 1500*g* for 15 minutes to obtain plasma, which was stored at –80°C.

### Immunophenotyping of Innate Immune Cells

We focused on innate immune cell phenotype and function, which are known to be impaired following sepsis^24^ and adapt to exposure to LPS and other stimuli.^25^ Our main read-outs were monocyte subpopulations, which are perturbed by systemic inflammation,^26^ and markers of monocyte antigen presenting capacity (HLA-DR); T-cell costimulation (CD86) and LPS responsiveness [Toll-like receptor (TLR)4, the LPS receptor]. We also assayed CD64, a high-affinity FcγRI membrane glycoprotein that mediates endocytosis, phagocytosis, antibody-dependent cellular toxicity, cytokine release and superoxide generation^27^; it is raised in both febrile viral and bacterial infections,^28^ and lower expression in the context of infections has been associated with increased mortality.^29^

**Figure 1:**
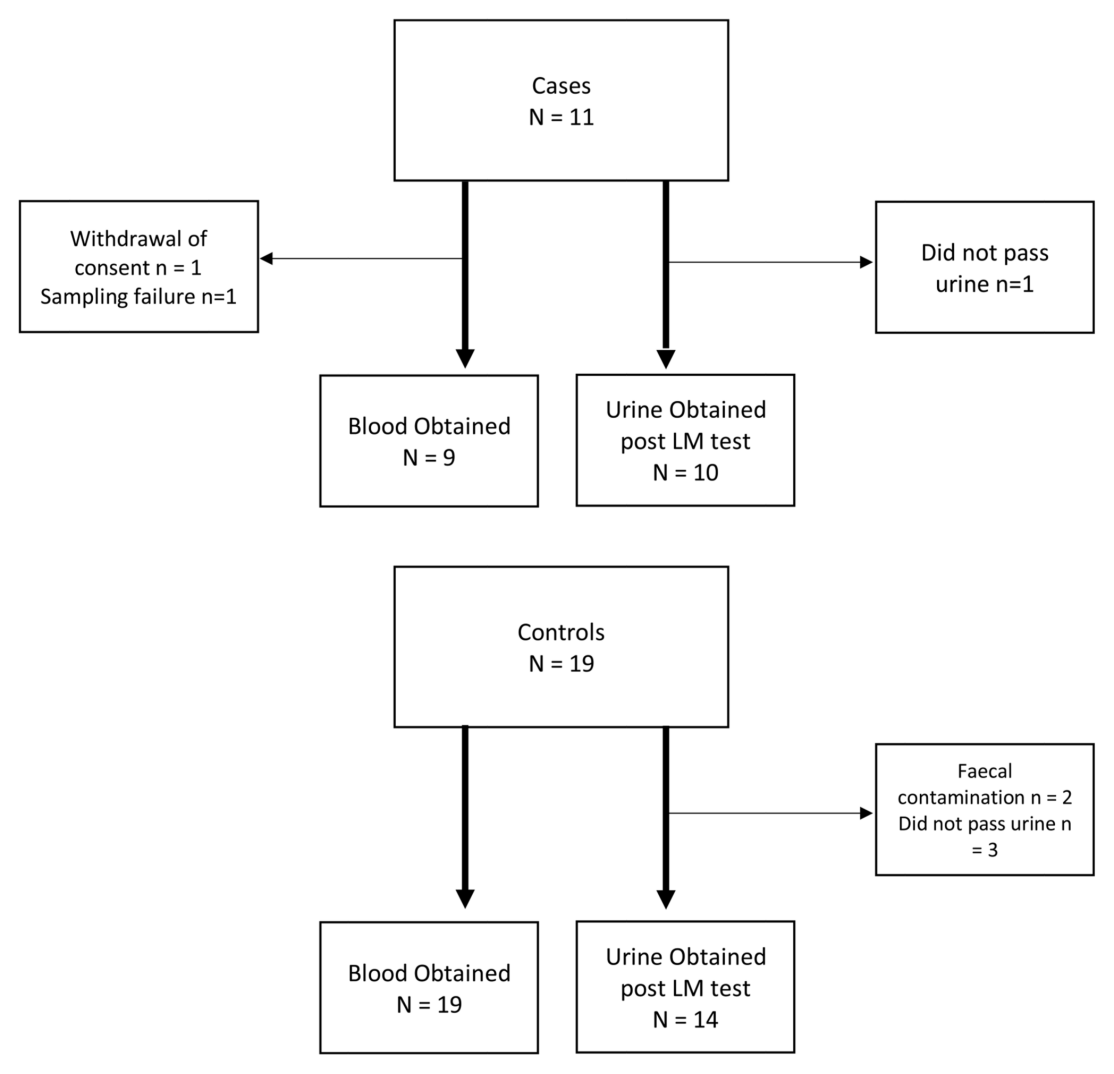
Number of children recruited to study and number of samples collected from them.

One hundred microliters of whole blood was stained for 30 minutes with fluorochrome-conjugated antibodies (CD14–PE; CD16–Pacific Blue; HLA-DR–APC/Fire; CD64–FITC; CD86–Brilliant Violet 605; TLR-4–APC and CD66a/c/e–PerCP-Cy5.5; all from Biolegend, San Diego, CA), following red-cell lysis (FACS Lysis Buffer, BD Biosciences, Franklin Lakes, NJ). The gating strategy used for identifying cell types and surface marker expression is shown in Fig. 1; Supplementary Digital Content 1, http://links.lww.com/INF/D447. Briefly, total monocytes were selected as the CD14+ CD66a/c/e-negative population and subtypes identified by their CD14 and CD16 expression as classical (CD14^++^ CD16^–^), intermediate (CD14^++^ CD16^+^) and nonclassical (CD14^+^ CD16^++^) monocytes.^30^ Neutrophils were identified as CD66a/c/e+ CD16^+^ cells.

Fluorescence-minus-one controls were used to set gating parameters. Compensation was undertaken with Ultracomp beads (eBioscience, San Diego, CA). Samples were run in duplicate on an LSR II (BD Biosciences) machine using BD FACSDiva software and analyzed using FlowJo v.10.2 software (FlowJo LLC, Ashland, OR).

### Twenty-four-hour Whole Blood Stimulation

Whole blood was diluted 6-fold using RPMI with 1% added penicillin–streptomycin, then 1 mL was cultured for 24 hours at 37°C with 5% CO_2_ in 24-well plates, stimulated in duplicate with 0.5 endotoxin unit (EU)/mL of LPS and 0.5 μg/mL of zymosan (both Sigma-Aldrich, St Louis, MO), or RPMI/1% penicillin–streptomycin without stimulant (control). After 24 hours, cells were analyzed by flow cytometry as described above, and culture supernatants were stored at –80°C for subsequent quantification of cytokines.

### Measurement of Cytokines

Plasma and cell culture supernatant cytokines were measured in duplicate using commercial ELISA kits following manufacturer’s instructions: interleukin 1 beta (IL-1β) and TNF-α (Duoset; R&D Systems, Minneapolis, MN, Golbal headquarters in USA as per comment in the questions), and IL-6 (OptEIA ELISA Kit II; BD Biosciences).

### Measurement of Endotoxin

Endotoxin concentrations were measured in plasma using a limulus amebocyte lysate assay (Pyrochrome kit; Associates of Cape Cod, Falmouth, MA) following the manufacturer’s instructions. Plasma was first diluted 1:10 with endotoxin-free water and then heated to 70°C for 30 minutes. The test was read using the kinetic method on a BioTek Synergy HT (Winooski, VT) plate reader. Detection limits were 0.01 EU/mL in undiluted plasma. Samples were analyzed in triplicate with the mean taken.

### Lactulose–Mannitol Assay

A lactulose–mannitol test was conducted on cases and controls as previously described.^31^ Briefly, a solution of 250 mg/mL lactulose and 50 mg/mL mannitol was given orally at a dose of 2 mL/kg up to a maximum of 20 mL. Children were fasted for at least 30 minutes and all urine was collected for 2 hours following administration of the solution. Aliquots of urine were stored at –80°C until analysis. Urinary lactulose and mannitol concentrations were measured using liquid chromatography-mass spectrometry, with a raffinose internal control. Each urine sample was analyzed twice and the mean value was used. A final lactulose:mannitol ratio (LMR) was calculated, using the recovery percentages of the lactulose and mannitol.

### Sample Size and Statistics

This was a pilot proof-of-concept study. We aimed to enroll as many cases and controls as possible between March 1, 2017 and June 1, 2017. Analysis was undertaken once all samples were collected and processed, using SPSS version 23.0 (IBM Corp, Armonk, NY) and Graphpad Prism v7.03 (GraphPad Software, San Diego, CA). Continuous variables were compared between groups using Mann-Whitney *U* tests, proportions were compared using Fisher Exact tests, and correlations were tested using Spearman rank correlation coefficients.

## Results

Thirty patients were recruited (11 cases, 19 controls), with baseline characteristics shown in Table 1. There were no significant differences between cases and controls in age, weight, or weight-for-age Z-scores. Among cases, children were diagnosed with lower respiratory tract infection (n = 4), skin abscesses (n = 3), urinary tract infection (treated as an ascending infection because of the fevers as per the national institute for health and care excellence guidelines; n = 2), osteomyelitis (n = 1) and sepsis with no source identified (n = 1). Among the noninfected controls, the majority (n = 13) were admitted for elective minor surgical day-case procedures including accessory digit removal, circumcision and dental procedures; others were admitted to the day-care unit for blood tests due to thalassemia (n = 5), or for consideration of a blood transfusion for sickle cell disease (n = 1). Due to difficulty sampling, not passing urine, or contamination of urine with feces, there were some missing samples, as shown in Figure 1.

### Cases Versus Controls

#### Intestinal Permeability and Circulating Endotoxin

**Figure 2:**
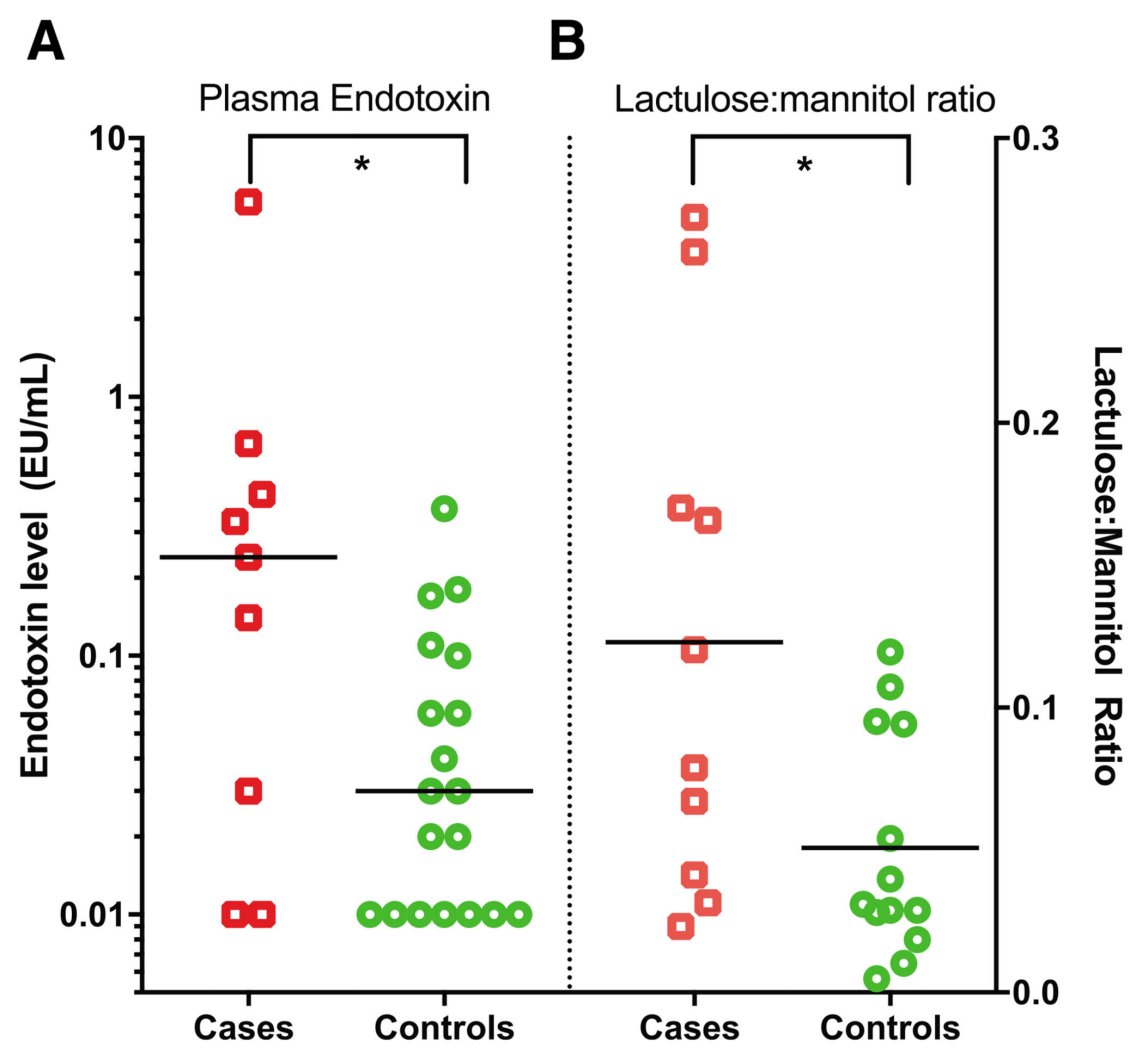
A, Circulating plasma endotoxin concentrations and (B) urine LMRs in children with noncritical infections (cases, red squares) and noninfected controls (green circles). The lowest level of endotoxin detection was 0.01 EU/mL. Horizontal bars indicate median values. Comparisons were made by Mann-Whitney *U* test; * = *P* < 0.05.

Plasma endotoxin concentrations were significantly higher in cases compared with controls (median 0.24 [interquartile range (IQR): 0.02, 0.54] vs. 0.02 (IQR: 0.01, 0.10) EU/mL, respectively; *P* = 0.028; Fig. 2A). Cases compared with controls also had increased intestinal permeability as measured by the LMR [median 0.09 (IQR: 0.03, 0.19) vs. 0.03 (IQR: 0.02, 0.09), respectively; *P* = 0.042; Fig. 2B]. This was due to both a decrease in mannitol recovery and an increase in lactulose recovery among cases (data not shown). There was a weak positive correlation between intestinal permeability and circulating endotoxin concentrations (Spearman coefficient *r* = 0.38, *P* = 0.086).

#### Monocyte and Neutrophil Phenotype

**Figure 3:**
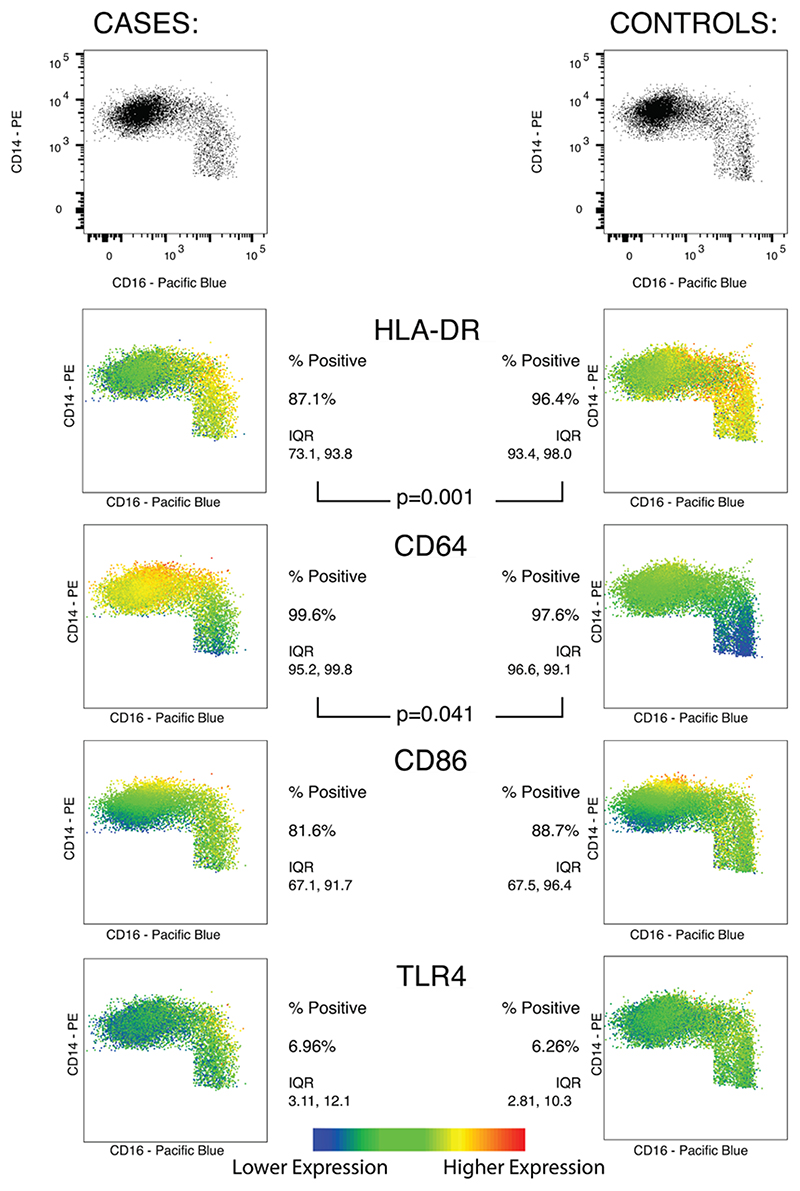
Percentage of total monocytes (CD66a/c/e– expressing HLA-DR, CD64, CD86 and TLR4 in cases and control groups, along with heat-maps showing the level of expression of each surface marker in each group according to the monocyte subpopulations defined according to CD14/16 expression: classical (CD14^++^ CD16^–^), intermediate (CD14^++^ CD16^+^) and nonclassical (CD14^+^ CD16^++^). Plots for each group shown are concatenated totals of all samples in the group. Comparisons made by Mann-Whitney *U* test.

There were no significant differences between the ratios of monocytes to lymphocytes between cases and controls (Table 1). Examining the peripheral blood monocyte population as a whole (CD66a/c/e– CD14+ cells), cases compared with controls had a significantly lower percentage expressing HLA-DR (median 87.1% vs. 96.4%; *P* = 0.001), and a higher percentage expressing CD64 (median 99.6% vs. 97.6%; *P* = 0.041, Fig. 3); there was no significant difference in the percentage of monocytes expressing CD86 or TLR4.

Among the monocyte subpopulations, there were no significant differences between cases and controls in proportions of classical [70.8% (IQR: 46.8, 78.0) vs. 72.6% (IQR: 58.4, 78.9), *P* = 0.56], intermediate [25.4% (IQR: 16.5, 43.8) vs. 20.2% (IQR: 16.2, 38.3), *P* = 0.74], or nonclassical [4.6% (IQR: 3.8 - 10.2) vs. 5.75% (4.6% - 7.5%), *P* = 1.00] monocytes. The HLA-DR percentage expression was significantly lower in cases compared with controls in both the classical [78.8% (IQR: 70.5, 93.0) vs. 95.9% (IQR: 92.0, 97.7), respectively; *P* = 0.001] and intermediate [91.4% (IQR: 76.3, 95.9) vs. 97.8% (IQR: 94.9, 98.8), *P* = 0.004] subpopulations but not the nonclassical (94.3% vs. 95.7%, *P* = 0.96) subpopulation. CD64 percentage expression on the nonclassical subpopulation was higher in cases than controls (88.9% vs. 67.0%, *P* = 0.014), but there was no evidence of difference between groups in CD64 expression on the classical (99.9% vs. 99.9%, *P* = 0.66) and intermediate (99.9% vs. 99.6%, *P* = 0.26) subpopulations (Fig. 3).

Finally, among the neutrophil population (CD66a/c/e+ CD16+), the percentage expression of CD64 was significantly higher in cases than controls [median 85.2% (IQR: 32.7, 94.7) vs. 26.9% (IQR: 9.81, 48.2); *P* = 0.025].

#### Twenty-four-hour Whole Blood Stimulation

**Figure 4:**
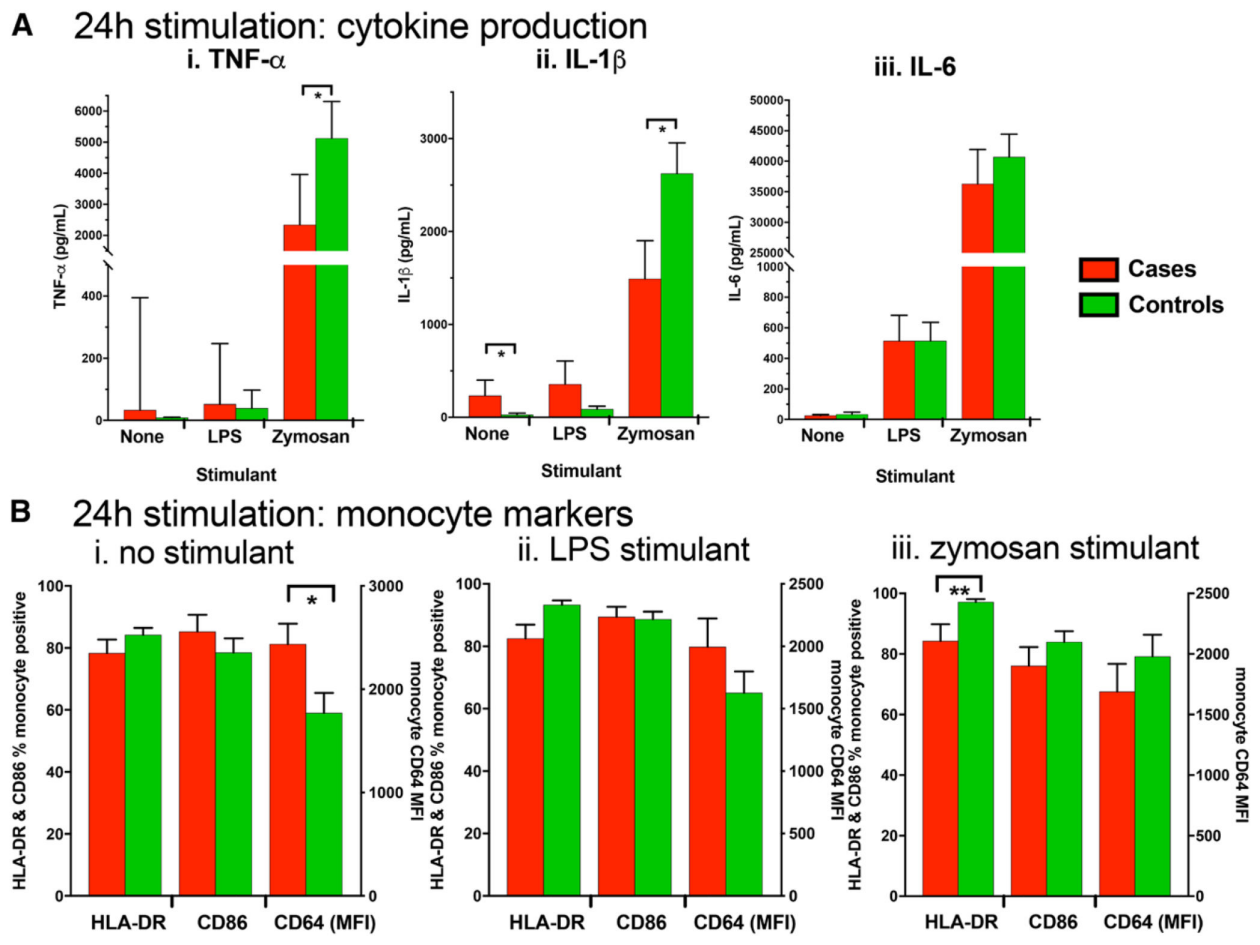
**A**, Whole blood cytokine production following 24-hour stimulation. **B**, Percentage of total monocytes (CD66a/c/ e–) expressing surface markers following whole blood culture with (i) no stimulation, (ii) 0.5 EU/mL of LPS or (iii) 0.5 μg/mL of zymosan. Comparisons were made by Mann-Whitney *U* test; * = *P*<0.05.

Concentrations of TNF-α, IL-1β and IL-6 measured in 24-hour whole blood culture supernatants are shown in Figure 4A. In unstimulated cultures, levels of IL-1β, but not IL-6 or TNF-α, were higher in cases versus controls. Stimulation with either 0.5 EU/mL of LPS or 0.5 μg/mL of zymosan resulted in significantly higher levels of all cytokines compared with cultures with no stimulation. Following stimulation with zymosan, cases versus controls produced significantly less TNF-α [median 2342 (IQR: 995, 3961) vs. 5130 (IQR: 2400, 6312) pg/mL, respectively; *P* = 0.031] and IL-1β [median 1101 (IQR: 564, 2313) vs. 2604 (IQR: 1339, 3528) pg/mL, *P* = 0.048], but similar IL-6 [median 36,716 (IQR: 18,830, 51,771) vs. 39,653 (IQR: 28,220, 52,695) pg/mL; *P* = 0.595]. Following stimulation with LPS, there was no evidence of a difference in cytokine production between cases and controls (Fig. 4A).

Percentage expression of cell surface markers on monocytes following 24 hours of culture is shown in Figure 4B. Because CD64 was universally expressed on monocytes following stimulation, the MFI was used to evaluate the level of CD64 expression on each cell. After 24-hour culture without stimulation, CD64 expression levels were higher among cases compared with controls [MFI 2570 (IQR: 1186, 2978) vs. 1591 (IQR: 950, 2173), respectively; *P* = 0.048; Fig. 4B], with no evidence of differences in other markers. Following stimulation with LPS, there was no evidence of differences between groups in the percentage of monocytes expressing any of the activation markers (Fig. 4B). Following stimulation with zymosan, the percentage of monocytes expressing HLA-DR in cases was significantly lower compared with controls [median 89.6% (IQR: 68.5, 97.0) vs. 99.0% (IQR: 97.4, 99.6), respectively; *P* = 0.009; Fig. 4B]. As has previously been observed,^1^ unstimulated monocyte HLA-DR expression across both groups was positively correlated with zymosan-stimulated TNF-α production (Spearman coefficient = 0.51, *P* = 0.006), and IL-1β (Spearman coefficient = 0.39, *P* = 0.041).

As expected, following 24-hour stimulation, there was a noticeable shift in the monocyte subpopulation proportions consistent with activation and maturation-specific shifts in CD14 and CD16 expression.^21^ The classical population was 13.6% (IQR: 4.8, 43.1) across all groups, the intermediate population was 81.3% (IQR: 57.1, 91.8) and the nonclassical was 2.6% (IQR: 0.7, 3.9). There was no significant difference in these shifts between cases and controls (data not shown).

## Low Versus High Endotoxin

To evaluate the impact of circulating endotoxin on monocyte immunophenotype, we combined cases and controls and categorized children into 2 groups: those with high circulating endotoxin (defined as ≥0.1 EU/mL), and those with low circulating endotoxin (<0.1 EU/mL). This categorization was based on a previous study, in which 0.1 EU/mL was identified as the upper normal limit in children, using the limulus amebocyte lysate assay.^32^ There were 11 children in the high endotoxin group [6 cases and 5 controls; median 0.18 (IQR: 0.11, 0.39) EU/mL endotoxin] and 17 in the low endotoxin group [3 cases and 14 controls; median 0.01 (IQR: 0.01, 0.025) EU/mL endotoxin].

### Monocyte Phenotype

**Figure 5:**
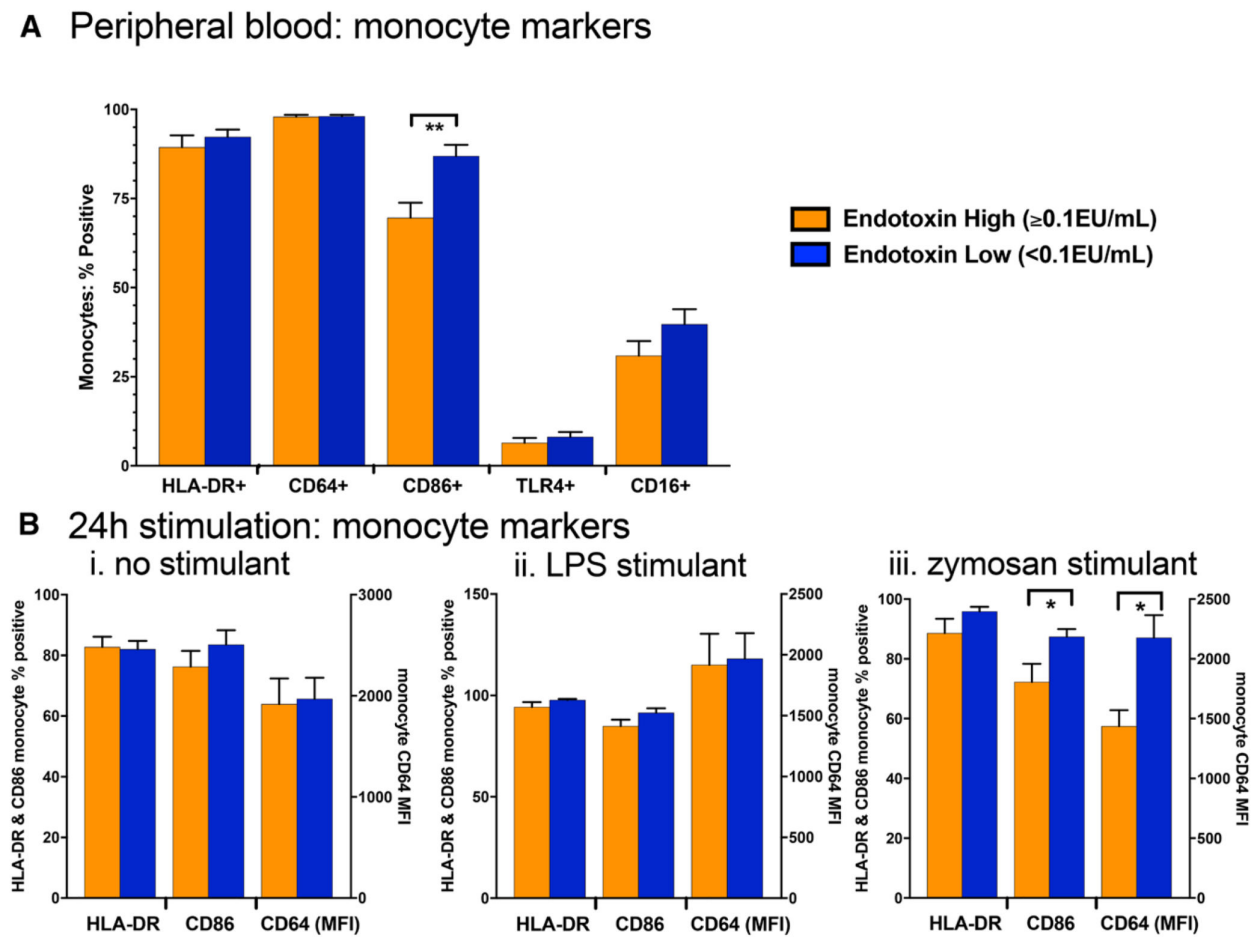
**A**, Surface marker expression on unstimulated monocytes (CD66a/c/e-) in cases and controls subdivided by high (>0.1 EU/mL) and low (<0.1 EU/mL) plasma endotoxin levels. **B**, Monocyte surface markers following 24-hour culture with (i) no stimulant, (ii) 0.5 EU/mL of LPS or (iii) 0.5 μg/mL of zymosan. Comparisons were made by Mann-Whitney *U* test; * =*P*<0.05.

In children with high compared with low endotoxin, the percentage of monocytes expressing the T-cell costimulatory ligand CD86 was significantly lower [69.8% (IQR: 58.8, 79.9) vs. 92.4% (IQR: 79.1, 96.4), *P* = 0.003; Fig. 5A]. There was no evidence of differences between groups in other markers (CD64, HLA-DR and TLR4). The lower CD86 expression on total monocytes among higher versus lower endotoxin was primarily due to lower expression on the classical monocytes [median 65.4% (IQR: 53.1, 78.3) vs. 87.2% (IQR: 76.1, 86.4); *P* = 0.08], and the intermediate monocytes [83.1% (IQR: 72.2, 92.0) vs. 96.6% (IQR: 84.8, 98.2); *P* = 0.13], but not on nonclassical monocytes [95.5% (IQR: 91.1, 97.9) vs. 96.0% (IQR: 86.3, 96.9); *P* = 0.41].

### Twenty-four-hour Whole Blood Stimulation

When comparing high and low endotoxin groups, there was no evidence of differences in elaboration of any cytokine in response to zymosan or LPS stimulation (data not shown). After stimulation with zymosan, children in the high compared with the low endotoxin group expressed significantly less CD64 [MFI 1514 (IQR: 1170, 1822) vs. 2196 (IQR: 1353, 2857), *P* = 0.022] and CD86 [80.3% (IQR: 57.2, 88.4) vs. 92.2% (IQR: 79.0, 96.0); *P* = 0.037] on total monocytes (Fig. 5B), with no evidence of differences in other markers.

## Discussion

In this study, we investigated whether the increased gut permeability and immunoparalysis that characterize critical illnesses also occur in children with less severe illnesses. We show that children with noncritical infections have higher intestinal permeability, increased endotoxemia and changes indicative of immunoparalysis, with reduced monocyte HLA-DR expression, increased CD64 expression and reduced IL-1β and TNF-α production upon challenge of innate immune cells with the beta-glucan, zymosan. In contrast to previous studies, children in this study were not critically unwell. Furthermore, we excluded children with any gastrointestinal symptoms to ensure that our findings were not due to altered intestinal permeability resulting from symptomatic intestinal infections. Endotoxemia as a result of increased intestinal permeability is thought to be a mediator of immunoparalysis in critically ill patients. In our cohort, children with higher circulating endotoxin levels had lower basal monocyte CD86 expression and less upregulation of CD64 and CD86 upon stimulation, indicative of an impaired capacity to costimulate T-cells and engage with antibody Fc components for enhanced specificity and recognition of pathogens. Collectively, these findings suggest that children hospitalized for noncritical infections experience changes in the intestinal barrier and circulating innate immune cell function normally associated with more critical illness, which could leave them vulnerable to subsequent infections.

The pathogenesis of increased intestinal permeability is likely multifactorial. In the context of gastrointestinal infections, there is mechanical damage to the epithelium, loss of tight junction integrity and villous atrophy arising from local inflammatory processes that cause cytokine-dependent changes in the enterocyte cytoskeleton.^33–35^ In critical illness or sepsis, there is hypoperfusion of the gut, which causes epithelial hypoxic injury, acidosis and disarrangement of the mucosal cytoskeleton.^35^ In noncritical infections outside the gut, where there is adequate gut perfusion, intestinal permeability is poorly described. However, increased intestinal permeability has been demonstrated in murine models of pneumonia,^36^ and in children with measles,^18^ although the presence of diarrhea in 30% of measles cases^37^ means the mechanism underlying this observation is difficult to ascertain. This study therefore adds to our understanding of gut function during intercurrent illness, because increased intestinal permeability was seen in young children with noncritical infections (predominantly in the skin, respiratory and urinary tract), and no gastrointestinal symptoms. In light of recent observations after experimental intravenous administration of LPS to healthy adult donors, which demonstrate a dramatic change in circulating monocyte phenotype and function,^19^ our data elaborate the association between intestinal permeability and monocyte function in children with noncritical illness, raising the possibility that their innate immune defences may also be compromised in ways previously thought to occur only in more severe illness.

Altered intestinal permeability can impair nutrient absorption, compromise mucosal immune responses^15,38^ and allow microbial products (such as endotoxin) and viable bacteria to translocate to the systemic circulation.^36^ Microbial translocation during critical illness was demonstrated clearly in an adult intensive care study: although only 4% of patients had Gram-negative infections, over half had endotoxin levels more than 2 standard deviations above healthy control levels.^5^ Although we cannot define the source of endotoxin in our study, it is likely that it arose from translocation of bacterial products across the intestinal barrier. This is an assumption made in other studies,^39,40^ confirmed in animal models^36^ and supported by the positive correlation between circulating endotoxin and intestinal permeability in our study. The weak positive relationship that we observed between LMR and circulating endotoxin has been found in a previous study,^41^ although others have found no correlation,^42,43^ or a relationship with the percentage lactulose recovery but not the LMR,^44^ albeit in very different populations. This heterogeneity may have several explanations: first, there may be a nonlinear relationship between permeability and translocation; second, it may reflect the fact that lactulose–mannitol testing primarily measures permeability of the small intestine, while the greatest Gram-negative bacterial load is in the colon^45^ and third, it may indicate that LPS is not the only translocated material that is likely to be immunogenic.

LPS-driven inflammatory responses are implicated in the pathogenesis of multiorgan dysfunction in systemic inflammatory response syndrome,^46^ and LPS exposure in monocytes is associated with epigenetic reprogramming leading to impaired responses on restimulation.^3,24^ Following LPS exposure in healthy adults, there is a rapid reduction in classical monocytes as these cells either die or likely mature into intermediate monocytes^21^ that have enhanced antigen presenting and stimulatory ability with increased HLA-DR^47^ and CD86 expression.^48^ The significance of this for our study is unclear: HLA-DR and CD64 percentage expression was associated with clinical illness rather than higher endotoxemia. This may reflect that the endotoxemia measured at the time cases were recruited may not reflect previous endotoxemia during their illness, and once exposed to LPS in vivo effects can last weeks, as opposed to the 24 hours seen in vitro models.^20^

Cases had lower HLA-DR and higher CD64 percentage expression than controls, but this was not due to altered numbers of intermediate monocytes; rather, cases had reduced HLA-DR expression on both classical and intermediate monocytes. The consequence of this reduced HLA-DR continued following 24 hours of culture with another stimulant, zymosan. There was a positive correlation between basal monocyte HLA-DR expression and overall TNF-α and IL-1β production following zymosan 24-hour culture, and both cytokines were significantly reduced in cases. This was not seen in IL-6 that is primarily produced by classical monocytes,^49^ which were significantly reduced relative to basal proportions by the end of the 24-hour culture. This suggests that any initial changes in circulating cell phenotype persist as monocytes mature from the classical to the predominantly intermediate subtypes in vitro. LPS-stimulated TNF-α production was modest and not significantly different between groups. This may reflect the fact that at 0.5 EU/mL, the concentration of LPS used for stimulation was not substantially higher than that detected in plasma of many of the patients. We did not see reversal of some of these changes, such as HLA-DR percentage, following culture with zymosan, as has previously been seen with beta-glucan stimulation of LPS-trained monocytes in vitro.^50^ Lower HLA-DR and CD64 expression on monocytes have been associated with increased mortality following sepsis,^29^ and along with reduced stimulated cytokine production, mirror the changes seen in postsepsis immunoparalysis.

In children with higher endotoxemia at the time of venepuncture, monocytes had significantly lower expression of CD86, which is a costimulatory molecule required for T-cell priming. Low CD86 expression on monocytes has been associated with more severe sepsis, as demonstrated by an increased Sequential Organ Failure Assessment score,^51^ and increased mortality in mouse models of sepsis.^52^ Monocytes from children with high plasma endotoxin in our study exhibited lower expression of CD64 and CD86 following stimulation with zymosan compared with monocytes from children with lower levels of circulating endotoxin, indicating that exposure to circulating LPS alters monocyte responses to subsequent pathogen challenge. These findings have been associated with mortality and poorer outcomes in children with sepsis.^29,52^

Limitations of this study include the small sample size, along with the heterogeneity of the etiology of the illnesses involved. This was intended to be a pilot study investigating responses to systemic infection; given some of the diagnostic uncertainty present in diagnosis in young children, we were able to show these changes across a variety of different types of infections. Because of different healthseeking behaviors of parents, cases presented at different timepoints in their illnesses, so the timing of blood tests was not standardized. Although it has been shown that monocyte markers do change over the course of an illness,^53^ in this study, the infected children were different to the controls regardless of illness timing. Finally, although the controls in this study did not have a febrile illness, they were still recruited from hospital, so would not necessarily be classed as “healthy.”

In summary, we show that children with noncritical infections and no gastrointestinal symptoms display similar gut and innate immune cell alterations as children who have severe sepsis or organ failure. In the setting of severe sepsis, these changes are associated with increased morbidity and mortality, and susceptibility to future infections. Future studies should investigate the molecular triggers of these observed changes, how long they persist and the clinical implications during and after hospitalization. Such studies are warranted because children with noncritical infections represent a much larger population of children presenting to hospital than children with critical illness. Whether there is a refractory period of immunoparalysis following common childhood infections, and whether interventions could improve immune function during this potential window of vulnerability, is worthy of further investigation.

## Supplementary Material

Supplementary Figures

## Figures and Tables

**Table 1:** Summary of Participants

	Cases (N = 11)	Controls (N = 19)	*P* Value (Cases vs. Controls)
Age, mo; median (range)	25 (6, 57)	31 (9, 52)	0.42*
Male, n (%)	7 (63.6)	12 (63.1)	0.45†
Weight, kg; median (range)	12.0 (9.1, 16.2)	13.4 (6.3, 27.0)	0.49*
Weight-for-age Z-score; median (range)	–0.15 (–0.77, 1.85)	–0.32 (–2.7, 3.2)	0.31*
Lymphocyte: monocyte ratio in whole blood; Median (range)	31.3 (7.4, 209)	21.2 (8.3, 67.7)	0.31*
CRP, mg/L; median (range)‡	109 (22, 236)	–	–
White cell count ×10^9^/L; median (range)‡	16.3 (6.9, 34.5)	–	–
Platelets × 10^9^/L; median (range)‡	279 (167, 440)	–	–

* Mann-Whitney ***U*** test.

† Fisher Exact test.

‡ CRP and differential cell counts were only measured in cases as part of their routine clinical care.
