# Supplementary material for: Children With Noncritical Infections Have Increased Intestinal Permeability, Endotoxemia and Altered Innate Immune Responses
Source: Pediatr Infect Dis J. Author manuscript; Available in PMC 2023 Aug 11. (PMC7614937; doi:10.1097/INF.0000000000002311)
Supplement: Supplementary Figures [file EMS184773-supplement-Supplementary_Figures.pdf]

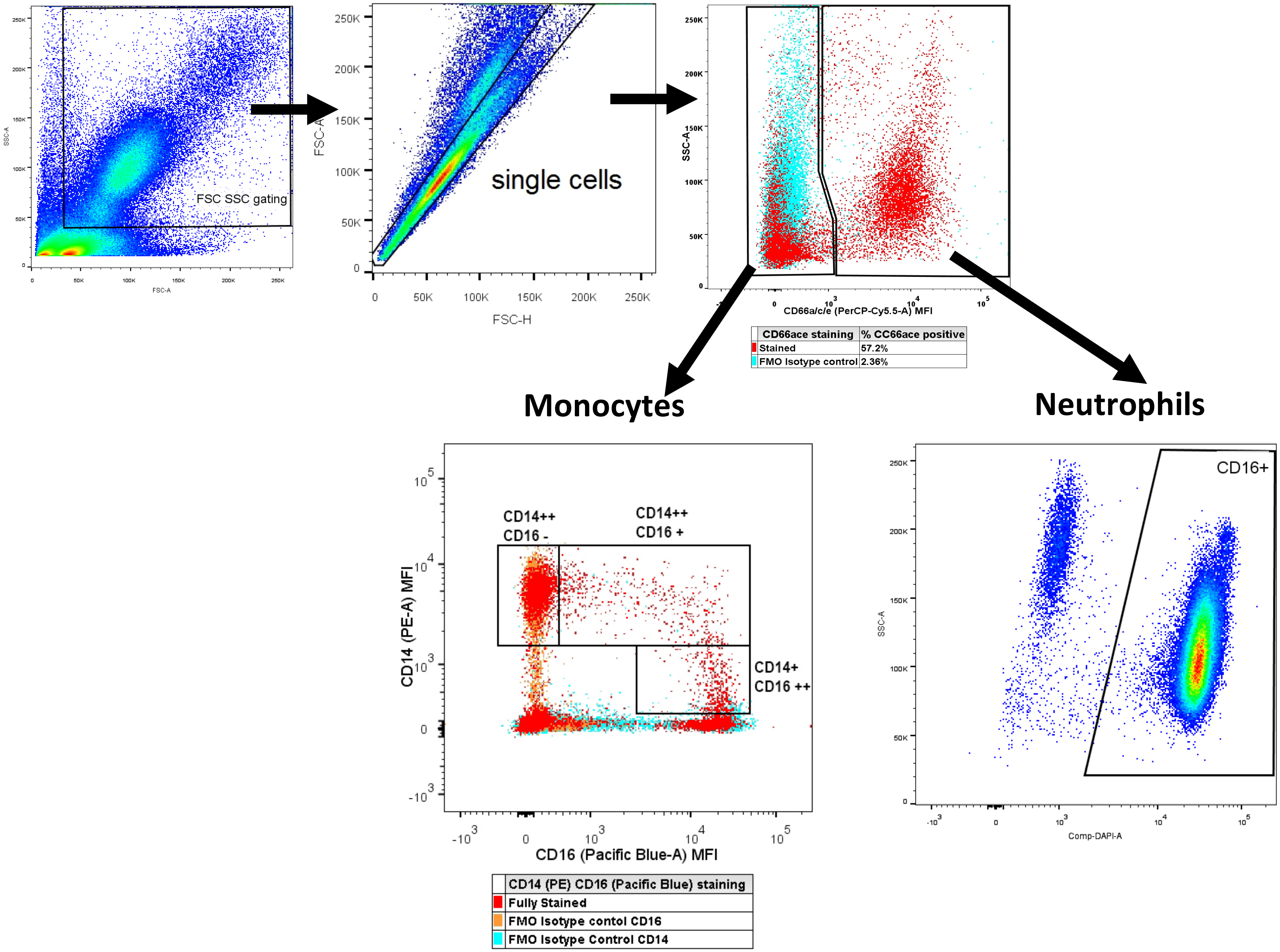
FSC SSC gating
SSC-A
FSC-A
FSC-A
FSC-H
single cells
SSC-A
CD66a/c/e (PerCP-Cy5.5-A) MFI
CD66ace staining | % CC66ace positive
Stained 57.2%
FMO Isotype control 2.36%
Monocytes
Neutrophils
CD14 (PE-A) MFI
CD16 (Pacific Blue-A) MFI
CD14++ CD16 -
CD14++ CD16 +
CD14+ CD16 ++
CD14 (PE) CD16 (Pacific Blue) staining
Fully Stained
FMO Isotype contol CD16
FMO Isotype Control CD14
CD16+
SSC-A
Comp-DAPI-A

Gating strategy of monocyte/neutrophil populations using FMO isotype controls:

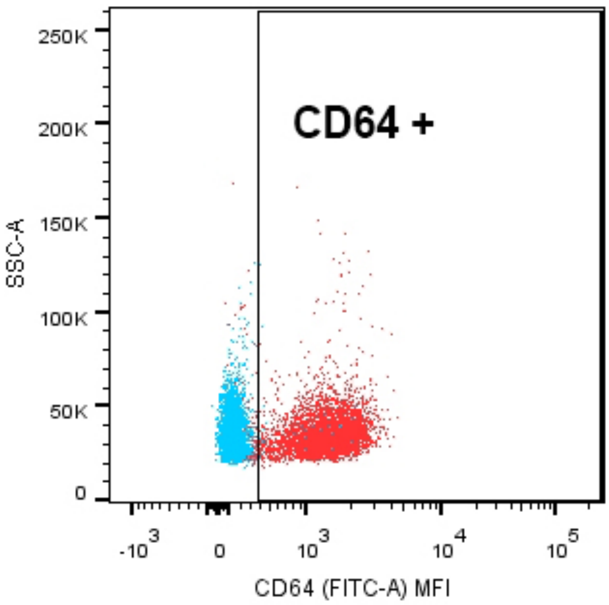

	CD64 (FITC) staining	% CD64 +ve
■ (blue)	FMO isotype control	0.65%
■ (red)	stained sample	96.4%

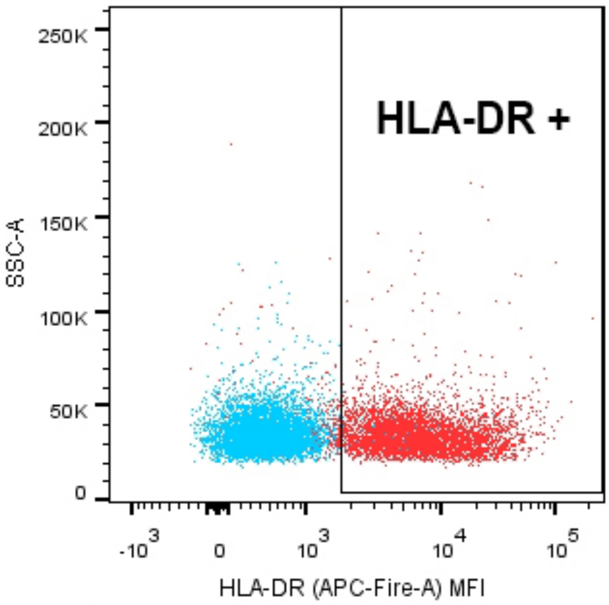

	HLA-DR (APC-Fire) staining	% HLA-DR positive
■ (blue)	FMO isotype control	0.34%
■ (red)	stained sample	92.8%

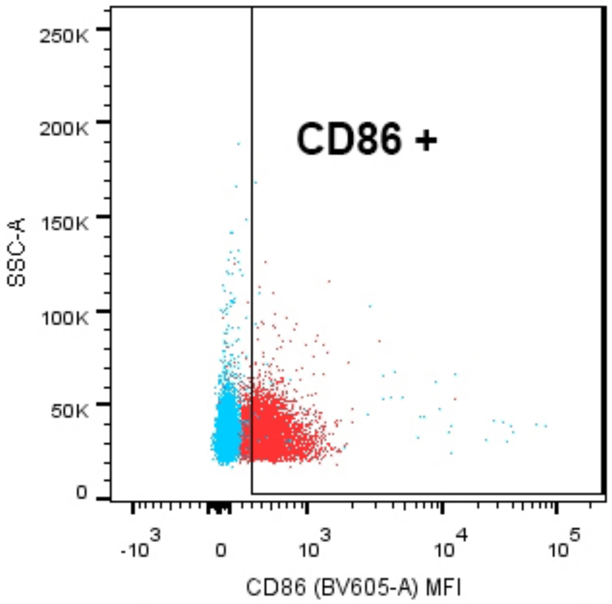

	CD86 (BV-605) staining	%CD86 positive
■ (blue)	FMO isotype control	0.73%
■ (red)	stained sample	67.3%

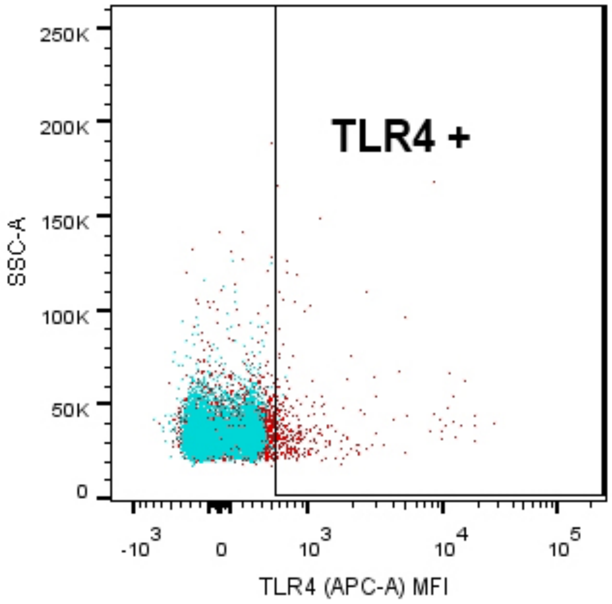

	TLR4 (APC) Staining	%TLR4 positive
■ (cyan)	FMO isotype control	0.15%
■ (dark red)	stained sample	4.21%
